# Choroid plexus in developmental and evolutionary perspective

**DOI:** 10.3389/fnins.2014.00363

**Published:** 2014-11-14

**Authors:** Brent Roy Bill, Vladimir Korzh

**Affiliations:** ^1^Semel Institute for Neuroscience and Human Behavior, University of California Los AngelesLos Angeles, CA, USA; ^2^Agency for Science, Technology and Research of Singapore, Institute of Molecular and Cell BiologySingapore, Singapore; ^3^National University of Singapore, Department of Biological SciencesSingapore, Singapore

**Keywords:** brain-CSF, transgenics, epithelium, vasculature, stroma, glia

## Abstract

The blood-cerebrospinal fluid boundary is present at the level of epithelial cells of the choroid plexus. As one of the sources of the cerebrospinal fluid (CSF), the choroid plexus (CP) plays an important role during brain development and function. Its formation has been studied largely in mammalian species. Lately, progress in other model animals, in particular the zebrafish, has brought a deeper understanding of CP formation, due in part to the ability to observe CP development *in vivo*. At the same time, advances in comparative genomics began providing information, which opens a possibility to understand further the molecular mechanisms involved in evolution of the CP and the blood-cerebrospinal fluid boundary formation. Hence this review focuses on analysis of the CP from developmental and evolutionary perspectives.

## What is the choroid plexus?

The choroid plexus (CP) is a set of ependymal-derived structures that regulate the composition of cerebrospinal fluid (CSF). Historically, the term “CP” (from Latin: chorion–delicate, plexus–knot) was defined by the vasculature; however, it now refers to multiple layers and cell types that form this organ (Strong, 1956; Netsky et al., 1975; Johanson, 2003; Redzic et al., 2005; Bill et al., 2008; Garcia-Lecea et al., 2008). The CP structure is conserved in vertebrate species, and consists of: a fenestrated vasculature core, an interstitial stromal layer, and a single layer of polarized cuboidal epithelia. Endothelial-derived and epithelial-derived basement membranes flank the stroma providing for a unique method of semipermeable filtration (Johanson et al., 2011). In most vertebrates there are four CP's: CPIII, CPIV, and two lateral CPs, inset within each ventricle. Due to differences in cortical development, teleosts have only two CPs, the diencephalic and myelencephalic CP (dCP and mCP) homologous with the midline CPs (CPIII and IV, respectively). For simplicity, we will primarily focus on comparisons between mCP and CPIV development.

Despite vital roles in development, physiology, and protection of the brain, the CP has remained outside of a sphere of interest for many mainstream neuroscientists. There are numerous reasons why this is the case including: reductionist approaches that refine work to a targeted neural tissue, a misconception that the CSF is only for metabolic support and waste removal, and a limited view of the pathologies that affect the CP. A renaissance in CP research has occurred over the past few years placing the CP as a critical modulator of the central nervous system elucidating roles during sleep, appetite, neural transmitter availability, and development of the brain (Zappaterra and Lehtinen, 2012). It has been implicated in over 30 multi-systemic disorders (Emerich et al., 2005; Lavezzi et al., 2013). Furthermore, it has presented itself as a potential target for pharmaceutical and cell-based treatments for Amblyopia, Stroke, Parkinson's disease, Huntington's disease, and Alzheimer's disease (Borlongan et al., 2004a,b; Emerich et al., 2006; Luo et al., 2013; Spatazza et al., 2013; Bolos et al., 2014). This clearly illustrates the need for a better understanding of the development and function of this vital organ.

A strong case can be made that the CP should be considered one of the circumventricular organs (Weindl and Joynt, 1973; Tsuneki, 1986; Ganong, 2000; Joly et al., 2007), as they have many similarities; for example, location at a periphery of the brain in close proximity to the ventricles, high levels of vascularization, functional roles in endocrine signaling, the production of proteins for secretion into the CSF, and the presence of fenestrated vessels (Molnar and Stolp; Richardson, this volume). However, the barrier function performed by the epithelia tight junctions of the CP has generated discussion as to its inclusion amongst circumventricular organs (Jeong et al., 2008; Umans and Taylor, 2012). Hence attempts to analyze the evolutionary and developmental origins of CP and some circumventricular organs such as subcommissural organ might shed light on this topic.

Comparative genomics early on demonstrated its importance for analysis of expression of imprinted genes in the developing CP (Overall et al., 1997), and genes critical for its development (Johansson et al., 2013). Recently, the detailed characterization of CP development in teleosts (Bill et al., 2008; Garcia-Lecea et al., 2008) and the significant progress in genome sequencing of model animals, including several fish (see, for example, Howe et al., 2013; Venkatesh et al., 2014) opened new horizons for advancement of comparative genomics of the CP on a much broader scale.

## Development of CP in mammals

In all vertebrates, the CPs develop in a posterior to anterior order with the CP of the IVth ventricle developing prior to more anterior CPs (Netsky et al., 1975; Knott et al., 1997; Garcia-Lecea et al., 2008). This is unlike many other developmental processes that progress from anterior to posterior, including, but not limited to, somitogenesis and neurodifferentiation (Korzh et al., 1993; Kimmel et al., 1995). It is tempting to link this sequence of events with other preceding neurodevelopmental events such as, closure of the neural tube in amphibians and mammals, which is a bidirectional process progressing both anteriorly and posteriorly from the hindbrain (Copp et al., 2003; Korzh, 2014).

The hindbrain roof plate epithelium has been considered to serve two major roles: the dorsal organizing center (Liem et al., 1997; Lee and Jessell, 1999; Lee et al., 2000; Millonig et al., 2000; Broom et al., 2012), and the source of the intermediate epithelium that transforms into cerebrospinal fluid-producing epithelium of the CPIV (Wilting and Christ, 1989; Thomas and Dziadek, 1993). As a dorsal organizing center, the hindbrain roof plate epithelium influences dorsal neuroectoderm, by secreting signaling molecules, such as BMPs and WNTs, to direct transcription in domains along the dorsal-ventral axis, specifically within the rhombic lip (Chizhikov and Millen, 2004; Currle et al., 2005). Reciprocal Notch signaling maintains the boundary between the hindbrain roof plate epithelium and the rhombic lip (Broom et al., 2012). Given that the cells of the CP epithelium and vascular are drawn to this location, it is tempting to hypothesize that the hindbrain roof plate epithelium secretes factors that recruit these cell types.

**Figure 1:**
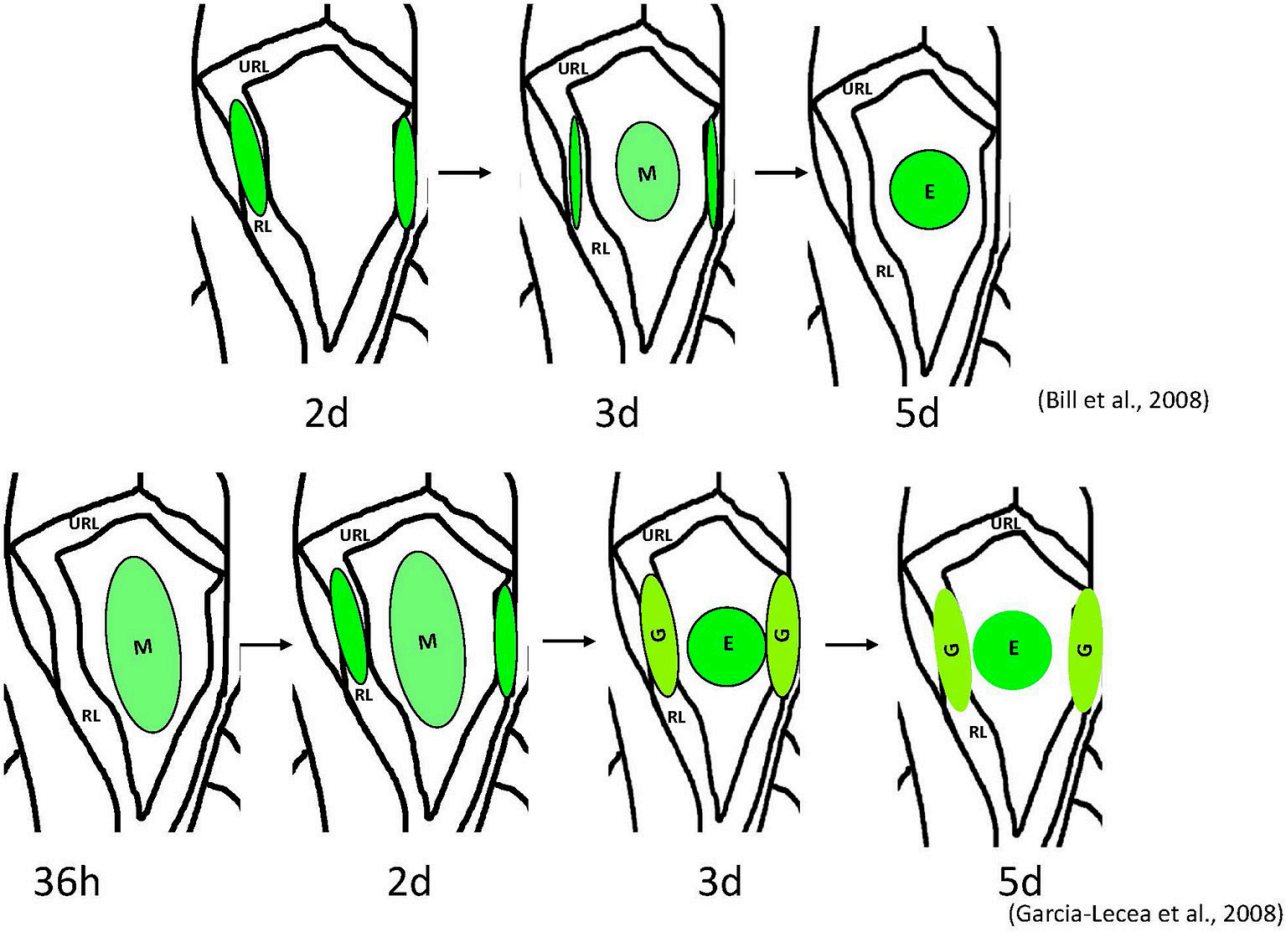
**Schemata of formation of the choroid plexus of IV ventricle in zebrafish as revealed by analysis of different transgenics**. Above– mn16Et, below – Gateways. The cytosolic GFP, first detected the lateral clusters, could be detected in the prospective epithelial cells of mn16Et at 3 dpf, i.e., after these coalesced toward the midline. This mimics events of the CP morphogenesis as hypothesized in mammals. In contrast, in the Gateway transgenics, the GFP is rather prominent in the midline cluster prior to its coalescence, i.e. during a stage of *tela choroidea*. Abbreviations: E, epidermal cells of the prospective choroid plexus of IV ventricle; G, glial cells of the prospective choroid plexus of IV ventricle; M, midline cluster; RL, rhombic lips; URL, upper rhombic lips.

It is thought that in mammals the hindbrain roof plate epithelium is primarily derived from the dorsal most neural ectoderm of the lateral rhombic lips, and these cells differ at the molecular level along the anterior-posterior and dorsal-ventral axes (Awatramani et al., 2003; Chizhikov et al., 2006; Hunter and Dymecki, 2007). The hindbrain roof plate epithelium consists of three distinct fields (Figure 1). The two primary fields are the medial and lateral fields, and they are distinct in developmental timing and mitotic activity. The lateral field expresses several molecular markers that further separate it into the lateral-anterior and lateral-posterior fields (Hunter and Dymecki, 2007). It appears that the lateral fields are the primary contributor of cells to CPIV, which initially develops as an invagination of the hindbrain roof plate epithelium (Awatramani et al., 2003). CPIV will subsequently receive migratory cells directly from the rhombic lip (Hunter and Dymecki, 2007). The fate-mapping studies of these fields lead to a number of questions. What is the fate of the midline field of hindbrain roof plate epithelium, and what are its contributions to CPIV development? Are the midline and lateral fields the sole source of cells present in the CPIV differentiated epithelium? Does the hindbrain roof plate epithelium also contribute to the CPIV vasculature or stroma? Is there a roof plate at the hindbrain (myelencephalon) level adjacent to the CPIV (mCP)? What genes are involved in specification of the different cell lineages in the CPIV? Are there cell adhesion molecules present on the cellular surface of developing CPIV cells that restrict cell type mixing, or do they segregate by developmental turnover as previously determined for the lateral CP (Liddelow et al., 2010)?

## Can fish provide information relevant to CP development in mammals?

**Figure 2:**
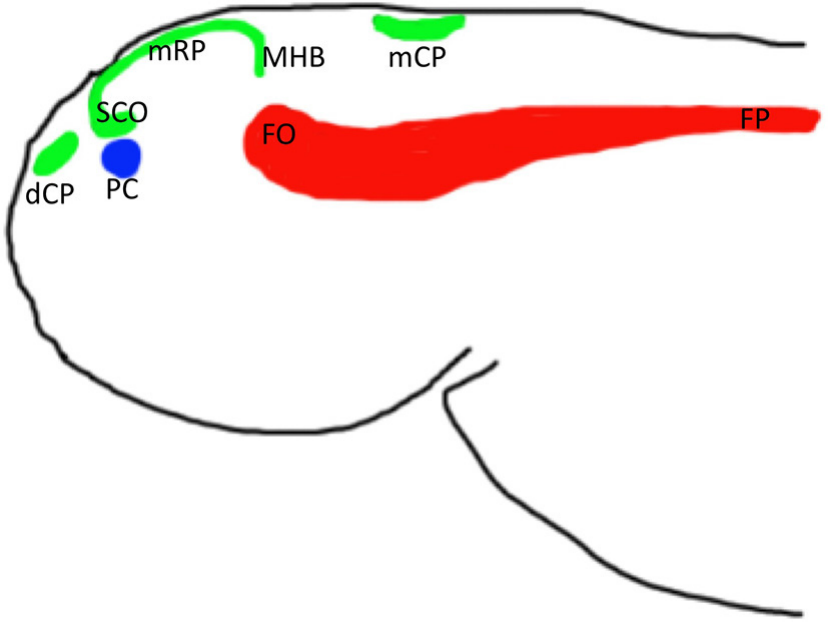
**Schemata of location of anterior midline structures of the neural tube in zebrafish based on analysis of several enhancer-trap transgenics**. Abbreviations: dCP, choroid plexus of diencephalon; mCP, choroid plexus of myelencephalon; FO, flexural organ; FP, floor plate; mRP, metencephalon (midbrain) roof plate; PC, posterior commissure; SCO, subcommissural organ; MHB, midbrain hindbrain boundary.

Given that all vertebrates, including fish have brain ventricles (Lowery and Sive, 2005), it is reasonable to expect that the molecular mechanisms underlying their formation were conserved during evolution, at least to some extent. For example, the Engrailed family of evolutionarily conserved transcriptional repressors plays an important role during formation of the midbrain-hindbrain boundary and adjacent tissues, the posterior midbrain and cerebellum. Engrailed loss-of-function mutations result in a reduction and/or loss of these structures, whereas gain-of-function mutations in Engrailed family members expressed at the dorsal midline cause misspecification of roof plate cells thus perturbing axonal navigation and interfering with the development of structures deriving from the dorsal neuroepithelium, such as the CP, epiphysis and subcommissural organ in fish, birds and rodents (Araki and Nakamura, 1999; Ristoratore et al., 1999; Louvi and Wassef, 2000). This suggests that the developmental program of the CP and some circumventricular organs is evolutionarily conserved opening a possibility to study these processes in the abundantly available and transparent embryos of fish (Figure 2). Indeed, the first *in vivo* studies of several enhancer-trap transgenic lines of zebrafish expressing cytosolic GFP (Table 1) brought about significant progress in understanding the development of CP.

**Table 1:** **Zebrafish transgenic lines that label the choroid plexus**.

**Transgenic line**	**Tissue**	**Genes linked**	**References**
sqet33Et	CP, dorsal interneurons, NCC, meninx	*zic3-zic6*	Kondrychyn et al., 2011; Winata et al., 2013
sqet3310Et	CP (epithelium), roof and floor plates, pigment and olfactory		Kondrychyn et al., 2011
sqet33e20Et (Gateways)	CP (medial and lateral clusters, tela choroidea, epithelium and glia), roof plate, NCC, CVOs, lens, pronephros, olfactory	*csrnp1b, sulf1, slc05A1*	Jiao et al., 2011; Kondrychyn et al., 2011
sqKR19Et	Expression pattern identical to that in SqET33-E20—CP (medial and lateral clusters, tela choroidea, epithelium and glia), roof plate, NCC, CVOs, lens, pronephros, olfactory	*sema3gb*	Teh et al., 2010
sqet33-mi84Et	CP (vasculature), general vasculature	*pard3*	Kondrychyn et al., 2011
sqgw18aEt	CP, roof plate, ear, eyes	Not mapped	Kondrychyn et al., 2011
sqgw19cEt	CP, roof plate, ventral neurons of spinal cord	Not mapped	Kondrychyn et al., 2011
sqgw42aEt	CP (epithelium), roof plate, ventral neurons	*csrnp1b, sulf1, slc05A1*	Garcia-Lecea et al., 2008
sqgw84bEt	CP, roof plate, pronephros, neurons	*slc22A13*	Kondrychyn et al., 2011
mn16Et	Lateral and medial clusters, tela choroidea, epithelium.	Not mapped	Bill et al., 2008

## The fate of the midline field and specification of CP lineages

As mentioned above, cell fate studies of CPIV in mice demonstrated a role of cells of the rhombic lips (lateral fields) in this process; however, the role of the hindbrain roof plate epithelium, in the absence of suitable markers, is not well-understood (Awatramani et al., 2003; Landsberg et al., 2005; Hunter and Dymecki, 2007). Interestingly, in zebrafish, a very similar developmental scenario can be proposed based upon observations of formation of the mCP. In the zebrafish transgenic line, mn16Et GFP expression is initially confined to the rhombic lips (lateral clusters), then spreads toward the midline. In time, only the midline cluster remains fluorescent (Bill et al., 2008). This could be interpreted in a manner similar to that in mice, i.e., that the lateral clusters of the rhombic lips are the only structures giving rise to the *tela choroidea*, the primordium of the CP.

Fortunately, zebrafish are amenable to large-scale genetic manipulation, thus additional *in vivo* markers for *tela choroidea* and CP were generated within our large-scale screen for enhancer-trap lines in zebrafish (Parinov et al., 2004; Kondrychyn et al., 2009). This provided the opportunity to study CP formation in real time. In particular, the transgenic line Gateways (sqet33e20Et) revealed formation of the dCP and mCP, each from several distinct cell groups, which is similar to that during formation of CPs in mice (Currle et al., 2005; Garcia-Lecea et al., 2008). The following phases of mCP formation were defined: (i) the *tela choroidea* forms from the neuroepithelial cells of the IVth ventricle roof, simultaneously the vasculature grows toward the final mCP location; (ii) some cells of the rhombic lips join the *tela choroidea* by migrating from the upper and lateral rhombic lips, reminiscent of the migration observed in mice and mn16Et zebrafish; (iii) the *tela choroidea* coalesces forming the button-like structure (medial cluster or mCP epithelial cells) in the roof of the IVth ventricle; (iv) the capillary encircles the structure and invades its interior; (v) as the *tela choroidea* coalesces, the remaining cells of the lateral clusters develop an astroglial phenotype, and (vi) these cells later converge with the medial cluster due to a reduction of the ventricle and ventricular roof. Therefore, this analysis revealed contribution into the mCP from at least four different cell lineages—the primary medial cluster derived from a roof of IVth ventricle, the secondary cells of medial cluster derived from lateral rhombic lips, the endothelial cells of the invading vasculature, and astroglial cells of the rhombic lips. Based on morphological criteria, the former two lineages contribute to the epithelial and stromal components of the CP, and the latter two correspond to endothelial and astroglial components. To support and develop this idea further more experimental evidence will be required. Nevertheless, based on unpublished results of other zebrafish transgenics, it is clear that these highlight events somewhat different from those seen in mn16Et and Gateways. It is critical that these transgenic and other developmental markers of CP be characterized, as they will further delineate unique contributions of different cell populations into the CP.

## Anterior roof plate: disappearing act or modifying morphogenesis?

The study of formation of the midline signaling structures in zebrafish transgenics revealed similar organization of the roof and floor plates along the spinal cord. During late neurulation, when the primitive lumen is reduced into a central canal, the roof plate is extended significantly along the dorsal-ventral axis, whereas floor plate is extended a little (Kondrychyn et al., 2013; Korzh, 2014). This organization reverses anteriorly, where starting from the myelencephalon level the floor plate progressively extends along the dorsal-ventral axis and the roof plate either is converted into other dorsal midline structures such as CP, or is significantly reduced as it happens at the level of mesencephalon (Korzh, 2007, 2014). Several observations support this interpretation. First, none of several roof plate transgenics analyzed so far express a fluorescent marker in the roof plate only; other expression domains represent the neural crest derivatives, interneurons, CP (see Table 1), i.e., structures derived from common progenitors (Papan and Campos-Ortega, 1999; Garcia-Lecea et al., 2008).

This raises an intriguing question: does the roof plate disappear partially and/or convert into other structures (CP or circumventricular organs)? Is this unique to bony fish? As mentioned above, there are several studies documenting the roof plate at the myelencephalon level in terrestrial vertebrates (Liem et al., 1997; Lee and Jessell, 1999; Lee et al., 2000; Millonig et al., 2000). Hence, the dorsal neural tube of bony fish may be evolutionary ancient and reminiscent of a common ancestor of vertebrates. And yet a presence of the most of circumventricular organs in zebrafish and terrestrial vertebrates (except those at the telencephalon level) makes this doubtful. One should not forget that current theories of formation of CP in bony fish and mammals still differ somewhat (Awatramani et al., 2003; Landsberg et al., 2005; Hunter and Dymecki, 2007; Korzh, 2007, 2014; Garcia-Lecea et al., 2008). Perhaps, the roof plate development in terrestrial vertebrates could be studied *in vivo*, at some point, or with help from comparative genomics, and results of such studies will contribute toward solving this interesting evolutionary puzzle.

Since the CP occupies a position at the dorsal midline, it is reasonable to predict that it forms by early neural progenitors found in this place. The roof plate occupies the dorsal midline of spinal cord. This poses a question, does the roof plate occupy the dorsal midline of the fourth (myelencephalic) ventricle, or do these cells somewhat differ? Dorsal neuroepithelium of the spinal cord prior to conversion into the roof plate undergoes topological transformation without cell migration. As a result the cells polarized initially along the medial-lateral axis become polarized along dorsal-ventral axis and elongate (Kondrychyn et al., 2013). In contrast, cells of midline dorsal neuroepithelium at the myelencephalic level fail to undergo such transformation. Instead, they coalesce into a cluster representing the epithelial lineage of CP. In parallel, the lateral clusters develop as glial cells morphologically reminiscent of the roof plate, but their presence is limited by an extent of 2–3 rhombomeres proximal to the medial cluster. These will fuse later on with the medial cluster as the glial lineage of the same CP (Garcia-Lecea et al., 2008). Each of these clusters represents approximately one half of the myelencephalic roof plate progenitors. Hence one may speculate that a ventricle separates the roof plate progenitors into two lateral clusters, and that the ventricle formation drives morphogenetic transformation of the dorsal midline progenitors into CP.

Hence at least for now there is no reason to believe that at the level of brain ventricles the roof plate is maintained. This consideration is supported by analysis of the roof plate formation in other brain regions, for example, the mesencephalon. At this level one more cavity reminiscent of a ventricle forms early on in parallel with the myelencephalon (hindbrain, IVth) and diencephalon (IIIrd) *bona fide* ventricles. This transient cavity known as the opthocoele develops an equivalent of the Sylvius aqueduct connecting two ventricles. At this level the roof plate cells are not as elongated as those at the spinal cord level, but they do have morphological similarity to the spinal cord roof plate. So, it seems we may formulate a simple hypothesis: “The roof plate is present at the dorsal midline of the neural tube only in absence of ventricles. In presence of ventricles it is converted into other derivatives, such as CP, etc.”

Does the roof plate contribute into any other midline derivatives? One of these is the subcommissural organ. It has been suggested that the subcommissural organ is derived from the neuroepithelial cells that line the lumen of the dorsal-caudal aspect of the diencephalon (Huh et al., 2009). In view of the fact that the subcommissural organ expresses several transgenes as the roof plate and/or CP, we suggest that this organ, located posterior and ventral to the epiphysis and, immediately below the posterior commissure, develops as the modified extension of the mesencephalic roof plate (Korzh, 2014).

## Genes of the dorsal midline

At the molecular level, there are at least two differences that have been identified in development of the CP in mammals and teleosts. First, *Otx2* null mice fail to develop all four CP; however, in teleosts, *otx2* expression appears limited to the diencephalon (Li et al., 1994; Johansson et al., 2013) thus potentially restricting its effects on the dCP. Despite several morpholinos designed for knockdown of Otx2 and available zebrafish mutants, the CPs in Otx2-deficient animals have not been investigated further. Second, transthyretinin (*TTR*), a common marker of the CP in amniotes, is not expressed in the CP of amphibians and teleost fish (Power et al., 2000). Given that in these species high levels of *ttr* are produced by the liver, future studies will be required to determine whether liver-derived *ttr* can functionally substitute for CP-derived *ttr*.

There are many more similarities in expression of molecular markers in the CP. Clusterin is the cytoprotective secreted chaperone protein linked to protection of cells from oxidative stress, and its deficiency has been implicated in Alzheimer's, metabolic and cardiovascular diseases (Trougakos, 2013; Park et al., 2014). Despite being vertebrate-specific, when expressed in *Drosophila*, Clusterin increases resistance to heat-shock, wet starvation, oxidative stress and increases the lifespan of flies (Lee et al., 2012). During mouse development its expression is enriched in the CP and ependyma (Charnay et al., 2008), and in zebrafish, it is expressed in developing mCP (Jiao et al., 2011). Clusterin may represent the earliest CSF protein secreted by the CP that suppresses proteotoxic stress. Clusterin expression is regulated by Notch signaling (Jiao et al., 2011). The developmental roles of both Notch and Shh signaling are conserved between fish and mammals, and Notch is known to regulate CP development (Hunter and Dymecki, 2007; Bill et al., 2008; Garcia-Lecea et al., 2008; Nielsen and Dymecki, 2010).

There are also several areas that will require more research to determine the similarities and differences in developmental activity of evolutionary conserved genes. The CPIII fails to develop in the Gli3-deficient mouse embryos (Grove et al., 1998). In zebrafish Gli3 is broadly expressed in the dorsal neural tube (Tyurina et al., 2005); however functional studies of CP in the Gli3-deficient zebrafish have not been performed. Amongst genes expressed in the CP of both mice and zebrafish, there are several Msx genes (Nishikawa et al., 1994; Landsberg et al., 2005; Thisse and Thisse, 2005); however, systematic comparisons will need to be performed and their specific roles in CP development established. Lastly, Gdf7 marks the primordium of the CP epithelium in mice, and has been utilized by many studies to drive expression within the CPIV (Currle et al., 2005); however, *gdf7* expression and function have not been analyzed in zebrafish in sufficient detail. These studies will be critical to determine the level of evolutionary conservation of developmental mechanisms of the CP and fill in a gap in this knowledge using molecular tools already available.

Translation of teleost findings to the mammalian systems could also be highly informative. The transposon-mediated enhancer-trap study identified several cell populations that contribute to the formation of the CP, and the insertion sites mapped identified the mCP-associated genes. In particular, the Gateway (sqet33e20Et) transgene has been mapped into a region containing *csrnp1a* (*axud1*), *sulf1*, and *slc5a1* (Garcia-Lecea et al., 2008). Transposon insertions of two other transgenic lines with expression in the CP (sqgw42aEt and sqgw45cEt) were mapped into the same genomic area supporting a role for one of these genes in the CP development or function. *csrnp1*, whose expression is controlled by Shh and PDGF, was proposed to be involved in regulating cell migration. It is essential for the development of neural-crest-derived cephalic structures (Schmahl et al., 2007; Feijoo et al., 2009). *slc5a1* (*sglt1*) has been studied for its role in glucose re-absorption in kidney and intestine (Gorboulev et al., 2012). Given that the functionally related glucose transporter 1 (GLUT1) is expressed in the CP of mammals, as well as that of bony and cartilaginous fish, this would make *slc5a1*, which encodes the sodium/glucose co-transporter rather interesting to investigate further despite the fact that its expression pattern might preclude a localized function (Farrell and Pardridge, 1991; Farrell et al., 1992; Planas et al., 2000; Jensen et al., 2010; Balmaceda-Aguilera et al., 2012). *sulf1* is expressed in the developing CP of mice (Ohto et al., 2002; Kalus et al., 2009; Ratzka et al., 2010), where its deficiency causes only minor defects or no defects in development of the CP probably due to functional redundancy with other Sulf genes (Holst et al., 2007; Kalus et al., 2009). Its orthologue in zebrafish is also expressed in the CP along with two other related genes—*sulf2* and *sulf2a* (Gorsi et al., 2010). Since *sulf1* has been linked to formation of the BBB, it is probably the best candidate gene, which expression is mimicked in the Gateways. Coincidentantly, the posttranslational modification of components of the extracellular matrix by sulfation plays an important role in regulation of cellular signaling (FGF, Hh, and Wnt) and mutations affecting components of sulfation machinery and its substrates are known to cause a variety of developmental defects linked to deficient cell signaling (Luders et al., 2003).

Lastly, several of the genes, specifically those that mediate the tight junctions between the CP epithelial cells are conserved across multiple phyla. These are not specific to the CP alone, but are a consistent feature between blood-CSF barrier, which is mediated at the level of epithelial cells of the CP (Dziegielewska et al., 2001); and BBB, which in all vertebrates is mediated at the level of endothelial cells of brain microvessels except sharks and rays, where it is at the level of astrocytes (Abbott et al., 2006; Bundgaard and Abbott, 2008). In the blood-CSF barrier the tight junction (or *zonula occludens*, ZO) is located in apical regions between adjacent epithelial cells, and filters the blood-to-CSF passage of hydrophilic molecules and ions (Johanson, 2003). Tight junctions are dynamic multimolecular complexes of membrane-associated proteins active in intracellular signaling, which include ZO proteins and claudins and a number of other proteins (Luissint et al., 2012). In the zebrafish there are three maternally-expressed genes encoding ZO proteins, so the ZO proteins are available from the very beginning of development (Kiener et al., 2007). Claudin 5a is also expressed in the brain during a period prior to and during CP formation (Zhang et al., 2010). Although a systematic analysis of genes expressed in the CP epithelia in evolutionary perspective is still in its infancy, there is a progress in this area. Hence, we will know to which extent the CP epithelium is conserved in evolution rather soon.

## Development of the CP vasculature

The vasculature of the human CPIV is supplied by branches that originate primarily from the posterior inferior cerebellar artery, and to a lesser extent the anterior inferior cerebellar artery (mostly in the anterior CP portion closest to the foramen of Lushka). In rare cases, branches of the superior cerebellar, the basilar artery, or other arteries extend branches to supply the CP (Sharifi et al., 2005). Once inside the CP the vessels become highly branched and interconnected providing a vast network. The vessels are unique from other brain regions in that they lack the endothelial barrier protein and astrocytic processes in the basement membrane (Bouchaud et al., 1989; Rosenstein et al., 1992), and contain 60–80 nm transcellular pores or fenestrations, allowing for the exit of substrates into the stroma (Strazielle and Ghersi-Egea, 2000; Stan et al., 2012). The fenestrations are covered by diaphragms that have tufts of heparan sulfate proteoglycans extending into the lumen suggesting a role in selective permeability (Stan et al., 2012). The dilation of these vessels is mediated by pericytes, innervating nerves, and interepithelial pressure sensors (Mortazavi et al., 2014), and has a direct effect on the composition of the CSF; therefore, they are highly regulated in response to CSF conditions independent of the physiological blood pressure (Scala et al., 2011). A specialized basement membrane, critical for serum filtering, is secreted by the vasculature very similar to that secreted by the fenestrated vasculature of the kidney (Johanson et al., 2011).

## Development of the mammalian CP vasculature

In human, the posterior inferior cerebellar artery and anterior inferior cerebellar artery begin sprouting at 44 and 40 days, respectively, and the posterior inferior cerebellar artery terminates in the CP at 52 days. Its arrival is concurrent with that of the first epithelial cells suggesting that signaling from the roof plate is critical for angiogenesis (Macchi et al., 2005). Development of the posterior inferior cerebellar artery is highly variable, with a variety of origins and paths consistent with a model in which hypoxia induces angiogenic factors, for example VEGF, and consequently angiogenic sprouting induced (Macchi et al., 2005; Yang et al., 2010). Angiogenic sprouting has been described in detail for the CP vasculature in other organisms as well. Strong described the process in 14-day rabbit embryos observing a reorganization of vessels occurring, with the pontobulbar artery running into the area that develops as the *tela choroidea* (Strong, 1956). Over the next few days the vasculature continues to develop, and anastomose being drawn into each progressive epithelial invagination (Strong, 1956). A second more rapid angiogenic process, called splitting angiogenesis or intussusceptive angiogenesis, is involved in the enumeration of vessels within the invaginations. This process was inferred from the identification of pillar structures bisecting larger vessels of the CP by electron microscopy (Scala et al., 2011). This developmental process has been primarily described in other tissues; however, it could be extrapolated to the CP vasculature. Intussusceptive angiogenesis describes three separate processes for vascular enumeration: microvascular growth, arborization, and remodeling (Makanya et al., 2009).

The zebrafish is one of the premiere animal models for studying vascular development due to its ability to develop without a functioning circulatory system or blood (Stainier, 2001), and large number of transgenics that label the vasculature (Lawson and Weinstein, 2002; Jin et al., 2005, 2007; Rehn et al., 2011; Trinh le et al., 2011; Zhou et al., 2011; Sacilotto et al., 2013). Unfortunately, the vasculature structure of the adult mCP has not been characterized as it has been in mammals. This is primarily due to an emphasis within the zebrafish community on early developmental processes, thus mCP characterization has been limited to the first 6 days post fertilization (dpf). Angiogenic sprouting occurs from the middle cerebral vessel at 48 hpf creating the dorsal longitudinal vein. The dorsal longitudinal vein extends along the dorsal midline turning at ~50 hpf to fuse with either left or right posterior cerebral vein (Bill et al., 2008; Lenard et al., 2013). The tip cells of the dorsal longitudinal vein and posterior cerebral vein initiate this junction, subsequently rearranging, and splitting to produce a multicellular lumen (Lenard et al., 2013). Consistent with events in the mammalian CPIV, the angiogenic sprout arrival occurs simultaneous to the migration of the epithelial field suggesting that the roof plate organizer is required to attract both sets of cells to their final localization. A secondary branch forms off of the main dorsal longitudinal vein branch and then fuses with the opposing posterior cerebral vein, and a third branch forms to connect the left and right posterior cerebral veins. We named this connecting vessel the trans-choroid plexus branch as it crosses near the posterior margin of mCP (Bill et al., 2008). Further branching between the dorsal longitudinal vein, posterior cerebral vein, and trans-choroid plexus branch are observed by 5 dpf internal to the mCP region defined by transgenic lines that label the mCP epithelia. The resulting structure is highly fragile and prone to hemorrhage (Bill, 2008) much like the mammalian CP (Scott and Bergevin, 2005). Given this fragility, it is not surprising, that the CP vasculature is constantly being repaired utilizing bone marrow-derived endothelial progenitors (Zhang et al., 2002). Unfortunately, processes such as invagination of the mCP, intussusceptive angiogenesis, and the integration of bone marrow-derived endothelial progenitors have not been studied in zebrafish. One striking difference is that the zebrafish appears to have a relatively invariant growth profile, while the vascular growth in humans is much more variable. There are two potential reasons for its invariant nature in the zebrafish. The first is that the corresponding period of zebrafish developmental program is much more rapid than that in human (2 h vs. 8 days). The increased developmental window could allow for competing attractive and repulsive angiogenic factors time to alter the trajectory of the main vessel. A second hypothesis could be related to the levels of oxygen that are driving vascular development. We know that the zebrafish are able to survive for up to 7 dpf without a functioning circulatory system, due to the permeability of the embryo to oxygen; therefore, hypoxic conditions within the developing brain would be much more common during human development. This would lead to involvement of competing angiogenic factors as other brain regions would attempt to obtain oxygen, and thus development would take a much more tortuous path.

At the molecular level sprouting angiogenesis, intussusceptive angiogenesis, and development of fenestrations are controlled by interactions between the VEGF, NOTCH, and SHH signaling pathways (Kamba et al., 2006; Nielsen and Dymecki, 2010; Yang et al., 2010; Dimova et al., 2013). The CP epithelial cells continuously secrete VEGF at high levels even in adults suggesting multiple roles for VEGF in vascular development (Esser et al., 1998), while the endothelial cells express the VEGF receptors. While these processes have been investigated extensively in the tail of the zebrafish, work on the cranial vasculature has lagged behind, and very little is known about the genes required for the dorsal longitudinal vein attraction to the mCP, branching, and production of the fenestrated vessels. Part of the problem is a general role of some of these pathways (Notch and Shh) in morphogenesis of the CP (Garcia-Lecea et al., 2008). In the future, extension of the developmental and molecular studies of the mCP vasculature will be required to determine the extent of conservation.

## Development of the CP stroma

The stroma lies between the vascular basement membrane and the epithelial basement membrane. It contains a wide variety of cells including: fibroblasts, telocytes, stem cells, dendritic cells, leukocytes, macrophages, kolmer cells (a specialized monocyte, also called epiplexus cells), and in some cases T-cells (Strazielle and Ghersi-Egea, 2000; Nataf et al., 2006; Popescu et al., 2012). The stroma is hypothesized to function as a stem cell niche with the nestin-positive fibroblasts and telocytes supporting myeloid progenitors and neural stem cells, respectively (Nataf et al., 2006; Popescu et al., 2012).

The stroma develops from invagination of the mesenchyme that lies dorsal to the hindbrain roof plate epithelium, and progressively transitions to being composed of high levels of connective tissues with low density of mesenchymal cells (Shuangshoti and Netsky, 1966). Little is known about the development of this region; however, there are indications that the stromal composition may be highly dynamic and change during development, and in response to immunologic insult. For example, it is hypothesized that the fibroblast niche provides different developmental signals during development than during adulthood based on the progeny of the myeloid progenitors; embryonic-derived progenitors produce dendritic cells, while adult-derived progenitors produce macrophages (Nataf et al., 2006). Another difference is that kolmer cells are only present in the adult CP (Strazielle and Ghersi-Egea, 2000). Given the immunologic complexity of the stroma, much of the cell diversity is driven by immunologic insult, or CNS injury (Mitchell et al., 2009; Wojcik et al., 2011; Hasegawa-Ishii et al., 2013; Shechter et al., 2013; Szmydynger-Chodobska et al., 2013). Previous analysis in zebrafish failed to demonstrate presence of unlabelled cells in the mCP of transgenics suggesting that all cell lineages except vasculature derive from GFP-positive cells (Garcia-Lecea et al., 2008). This raises a criticism regarding contribution of mesenchymal cells into the mCP of this species; however, as mentioned all analyses on zebrafish have focused on early development opening the possibility that the mesenchymal-derived cells enter at later developmental time points. New *in vitro* systems and transgenics in combination with high-resolution bioimaging may lead to a much better definition of this portion of the mCP.

## Conclusions

The CP has been a popular organ system to investigate in a variety of vertebrates including fish, lizards, amphibians, birds, and mammals; however, the number of systematic studies that look at a single feature of the CP across the phyla have been very few, and primarily restricted to the CP epithelia. Given the CP's importance in a variety of physiologic brain functions and pathologies, we suggest a need for these types of studies, as they provide valuable insights into understanding CP development and function. In this review, we have tried to highlight how several studies in the zebrafish have expanded our knowledge of CP development in general, and shed light on several aspects that remain not fully understood, including developmental fate of the medial group of hindbrain roof cells. Based on comparisons of fish and mice, we observe that the CP derives from cells that share their origin with the roof plate and neural crest, and its development is driven by the evolutionary conserved molecular determinants. Indeed, this is a renaissance in CP research, and there are many questions that are yet to be answered. For example, what are the tropic factors that are secreted from the dorsal organizing center to attract the vasculature and epithelia cells to their final location? What are the molecular differences that exist within the CP, as cells can be derived from either the roof plate or neural crest? What is the molecular identity of the stem cell niche? And lastly, to what extent are features of the CP conserved? Given an abundance of modern developmental tools (transgenics and cell transplantation), and genomic technologies (single cell transcriptome and genome engineering), we are poised to answer these questions, and feel that they can be answered soon.

### Conflict of interest statement

The authors declare that the research was conducted in the absence of any commercial or financial relationships that could be construed as a potential conflict of interest.

## References

[B83] Abbott N. J. Ronnback L. Hansson E. (2006). Astrocyte-endothelial interactions at the blood-brain barrier. Nat. Rev. Neurosci. 7, 41–53. 10.1038/nrn1824 16371949

[B46] Araki I. Nakamura H. (1999). Engrailed defines the position of dorsal di-mesencephalic boundary by repressing diencephalic fate. Development 126, 5127–5135. 10529429 10.1242/dev.126.22.5127

[B41] Awatramani R. Soriano P. Rodriguez C. Mai J. J. Dymecki S. M. (2003). Cryptic boundaries in roof plate and choroid plexus identified by intersectional gene activation. Nat. Genet. 35, 70–75. 10.1038/ng1228 12923530

[B71] Balmaceda-Aguilera C. Cortes-Campos C. Cifuentes M. Peruzzo B. Mack L. Tapia J. C.. (2012). Glucose transporter 1 and monocarboxylate transporters 1, 2, and 4 localization within the glial cells of shark blood-brain-barriers. PLoS ONE 7:e32409. 10.1371/journal.pone.0032409 22389700 PMC3289654

[B107] Bill B. R. (2008). Utilization of Forward and Reverse Genetic Approaches to Inform Ocular and Choroid Plexus Development. Molecular, Cellular, Developmental Biology, and Genetics. Minneapolis, MN: University of Minnesota.

[B1] Bill B. R. Balciunas D. McCarra J. A. Young E. D. Xiong T. Spahn A. M.. (2008). Development and Notch signaling requirements of the zebrafish choroid plexus. PLoS ONE 3:e3114. 10.1371/journal.pone.0003114 18769591 PMC2528000

[B13] Bolos M. Antequera D. Aldudo J. Kristen H. Bullido M. J. Carro E. (2014). Choroid plexus implants rescue Alzheimer's disease-like pathologies by modulating amyloid-beta degradation. Cell. Mol. Life Sci. 71, 2947–2955. 10.1007/s00018-013-1529-4 24343520 PMC11113864

[B14] Borlongan C. V. Skinner S. J. Geaney M. Vasconcellos A. V. Elliott R. B. Emerich D. F. (2004a). Intracerebral transplantation of porcine choroid plexus provides structural and functional neuroprotection in a rodent model of stroke. Stroke 35, 2206–2210. 10.1161/01.STR.0000138954.25825.0b 15284450

[B15] Borlongan C. V. Skinner S. J. Geaney M. Vasconcellos A. V. Elliott R. B. Emerich D. F. (2004b). Neuroprotection by encapsulated choroid plexus in a rodent model of Huntington's disease. Neuroreport 15, 2521–2525. 10.1097/00001756-200411150-00018 15538187

[B90] Bouchaud C. Le Bert M. Dupouey P. (1989). Are close contacts between astrocytes and endothelial cells a prerequisite condition of a blood-brain barrier? The rat subfornical organ as an example. Biol. Cell 67, 159–165. 2698759

[B36] Broom E. R. Gilthorpe J. D. Butts T. Campo-Paysaa F. Wingate R. J. (2012). The roof plate boundary is a bi-directional organiser of dorsal neural tube and choroid plexus development. Development 139, 4261–4270. 10.1242/dev.082255 23052907 PMC3478690

[B84] Bundgaard M. Abbott N. J. (2008). All vertebrates started out with a glial blood-brain barrier 4-500 million years ago. Glia 56, 699–708. 10.1002/glia.20642 18338790

[B61] Charnay Y. Imhof A. Vallet P. G. Hakkoum D. Lathuiliere A. Poku N.. (2008). Giannakopoulos, Clusterin expression during fetal and postnatal CNS development in mouse. Neuroscience 155, 714–724. 10.1016/j.neuroscience.2008.06.022 18620027

[B39] Chizhikov V. V. Millen K. J. (2004). Mechanisms of roof plate formation in the vertebrate CNS. Nat. Rev. Neurosci. 5, 808–812. 10.1038/nrn1520 15378040

[B42] Chizhikov V. V. Lindgren A. G. Currle D. S. Rose M. F. Monuki E. S. Millen K. J. (2006). The roof plate regulates cerebellar cell-type specification and proliferation. Development 133, 2793–2804. 10.1242/dev.02441 16790481

[B30] Copp A. J. Greene N. D. Murdoch J. N. (2003). The genetic basis of mammalian neurulation. Nat. Rev. Genet. 4, 784–793. 10.1038/nrg1181 13679871

[B40] Currle D. S. Cheng X. Hsu C. M. Monuki E. S. (2005). Direct and indirect roles of CNS dorsal midline cells in choroid plexus epithelia formation. Development 132, 3549–3559. 10.1242/dev.01915 15975937

[B111] Dimova I. Hlushchuk R. Makanya A. Styp-Rekowska B. Ceausu A. Flueckiger S.. (2013). Inhibition of Notch signaling induces extensive intussusceptive neo-angiogenesis by recruitment of mononuclear cells. Angiogenesis 16, 921–937. 10.1007/s10456-013-9366-5 23881168

[B82] Dziegielewska K. M. Ek J. Habgood M. D. Saunders N. R. (2001). Development of the choroid plexus. Microsc Res. Tech. 52, 5–20. 10.1002/1097-0029(20010101)52:1<5::AID-JEMT3>3.0.CO;2-J 11135444

[B9] Emerich D. F. Skinner S. J. Borlongan C. V. Vasconcellos A. V. Thanos C. G. (2005). The choroid plexus in the rise, fall and repair of the brain. Bioessays 27, 262–274. 10.1002/bies.20193 15714561

[B16] Emerich D. F. Thanos C. G. Goddard M. Skinner S. J. Geany M. S. Bell W. J.. (2006). Extensive neuroprotection by choroid plexus transplants in excitotoxin lesioned monkeys. Neurobiol. Dis. 23, 471–480. 10.1016/j.nbd.2006.04.014 16777422

[B112] Esser S. Wolburg K. Wolburg H. Breier G. Kurzchalia T. Risau W. (1998). Vascular endothelial growth factor induces endothelial fenestrations *in vitro*. J. Cell Biol. 140, 947–959. 10.1083/jcb.140.4.947 9472045 PMC2141756

[B72] Farrell C. L. Pardridge W. M. (1991). Blood-brain barrier glucose transporter is asymmetrically distributed on brain capillary endothelial lumenal and ablumenal membranes: an electron microscopic immunogold study. Proc. Natl. Acad. Sci. U.S.A. 88, 5779–5783. 10.1073/pnas.88.13.5779 2062858 PMC51961

[B73] Farrell C. L. Yang J. Pardridge W. M. (1992). GLUT-1 glucose transporter is present within apical and basolateral membranes of brain epithelial interfaces and in microvascular endothelia with and without tight junctions. J. Histochem. Cytochem. 40, 193–199. 10.1177/40.2.1552163 1552163

[B68] Feijoo C. G. Sarrazin A. F. Allende M. L. Glavic A. (2009). Cystein-serine-rich nuclear protein 1, Axud1/Csrnp1, is essential for cephalic neural progenitor proliferation and survival in zebrafish. Dev. Dyn. 238, 2034–2043. 10.1002/dvdy.22006 19544579

[B17] Ganong W. F. (2000). Circumventricular organs: definition and role in the regulation of endocrine and autonomic function. Clin. Exp. Pharmacol. Physiol. 27, 422–427. 10.1046/j.1440-1681.2000.03259.x 10831247

[B2] Garcia-Lecea M. Kondrychyn I. Fong S. H. Ye Z. R. Korzh V. (2008). *In vivo* analysis of choroid plexus morphogenesis in zebrafish. PLoS ONE 3:e3090. 10.1371/journal.pone.0003090 18769618 PMC2525818

[B70] Gorboulev V. Schurmann A. Vallon V. Kipp H. Jaschke A. Klessen D.. (2012). Na(+)-D-glucose cotransporter SGLT1 is pivotal for intestinal glucose absorption and glucose-dependent incretin secretion. Diabetes 61, 187–196. 10.2337/db11-1029 22124465 PMC3237647

[B80] Gorsi B. Whelan S. Stringer S. E. (2010). Dynamic expression patterns of 6-O endosulfatases during zebrafish development suggest a subfunctionalisation event for sulf2. Dev. Dyn. 239, 3312–3323. 10.1002/dvdy.22456 20981828

[B64] Grove E. A. Tole S. Limon J. Yip L. Ragsdale C. W. (1998). The hem of the embryonic cerebral cortex is defined by the expression of multiple Wnt genes and is compromised in Gli3-deficient mice. Development 125, 2315–2325. 9584130 10.1242/dev.125.12.2315

[B116] Hasegawa-Ishii S. Shimada A. Inaba M. Li M. Shi M. Kawamura N.. (2013). Selective localization of bone marrow-derived ramified cells in the brain adjacent to the attachments of choroid plexus. Brain Behav. Immun. 29, 82–97. 10.1016/j.bbi.2012.12.010 23270678

[B79] Holst C. R. Bou-Reslan H. Gore B. B. Wong K. Grant D. Chalasani S.. (2007). Secreted sulfatases Sulf1 and Sulf2 have overlapping yet essential roles in mouse neonatal survival. PLoS ONE 2:e575. 10.1371/journal.pone.0000575 17593974 PMC1892809

[B25] Howe K. Clark M. D. Torroja C. F. Torrance J. Berthelot C. Muffato M.. (2013). The zebrafish reference genome sequence and its relationship to the human genome. Nature 496, 498–503. 10.1038/nature12111 23594743 PMC3703927

[B55] Huh M. S. Todd M. A. Picketts D. J. (2009). SCO-ping out the mechanisms underlying the etiology of hydrocephalus. Physiology 24, 117–126. 10.1152/physiol.00039.2008 19364914

[B43] Hunter N. L. Dymecki S. M. (2007). Molecularly and temporally separable lineages form the hindbrain roof plate and contribute differentially to the choroid plexus. Development 134, 3449–3460. 10.1242/dev.003095 17728348 PMC2897145

[B74] Jensen P. J. Gunter L. B. Carayannopoulos M. O. (2010). Akt2 modulates glucose availability and downstream apoptotic pathways during development. J. Biol. Chem. 285, 17673–17680. 10.1074/jbc.M109.079343 20356836 PMC2878531

[B21] Jeong J. Y. Kwon H. B. Ahn J. C. Kang D. Kwon S. H. Park J. A.. (2008). Functional and developmental analysis of the blood-brain barrier in zebrafish. Brain Res. Bull. 75, 619–628. 10.1016/j.brainresbull.2007.10.043 18355638

[B62] Jiao S. Dai W. Lu L. Liu Y. Zhou J. Li Y.. (2011). The conserved clusterin gene is expressed in the developing choroid plexus under the regulation of notch but not IGF signaling in zebrafish. Endocrinology 152, 1860–1871. 10.1210/en.2010-1183 21385939

[B99] Jin S. W. Beis D. Mitchell T. Chen J. N. Stainier D. Y. (2005). Cellular and molecular analyses of vascular tube and lumen formation in zebrafish. Development 132, 5199–5209. 10.1242/dev.02087 16251212

[B100] Jin S. W. Herzog W. Santoro M. M. Mitchell T. S. Frantsve J. Jungblut B.. (2007). A transgene-assisted genetic screen identifies essential regulators of vascular development in vertebrate embryos. Dev. Biol. 307, 29–42. 10.1016/j.ydbio.2007.03.526 17531218 PMC2695512

[B3] Johanson C. (2003). The choroid plexus—CSF nexus, in Neuroscience in Medicine, ed Conn P. M. (Totowa, NJ: Humana Press), 165–195.

[B7] Johanson C. E. Stopa E. G. McMillan P. N. (2011). The blood-cerebrospinal fluid barrier: structure and functional significance. Methods Mol. Biol. 686, 101–131. 10.1007/978-1-60761-938-3_4 21082368

[B24] Johansson P. A. Irmler M. Acampora D. Beckers J. Simeone A. Gotz M. (2013). The transcription factor Otx2 regulates choroid plexus development and function. Development 140, 1055–1066. 10.1242/dev.090860 23364326

[B18] Joly J. S. Osorio J. Alunni A. Auger H. Kano S. Retaux S. (2007). Windows of the brain: towards a developmental biology of circumventricular and other neurohemal organs. Semin. Cell Dev. Biol. 18, 512–524. 10.1016/j.semcdb.2007.06.001 17631396

[B76] Kalus I. Salmen B. Viebahn C. von Figura K. Schmitz D. D'Hooge R.. (2009). Differential involvement of the extracellular 6-O-endosulfatases Sulf1 and Sulf2 in brain development and neuronal and behavioural plasticity. J. Cell. Mol. Med. 13, 4505–4521. 10.1111/j.1582-4934.2008.00558.x 20394677 PMC4515066

[B110] Kamba T. Tam B. Y. Hashizume H. Haskell A. Sennino B. Mancuso M. R.. (2006). VEGF-dependent plasticity of fenestrated capillaries in the normal adult microvasculature. Am. J. Physiol. Heart Circ. Physiol. 290, H560–H576. 10.1152/ajpheart.00133.2005 16172168

[B86] Kiener T. K. Sleptsova-Friedrich I. Hunziker W. (2007). Identification, tissue distribution and developmental expression of tjp1/zo-1, tjp2/zo-2 and tjp3/zo-3 in the zebrafish, *Danio rerio*. Gene Expr. Patterns 7, 767–776. 10.1016/j.modgep.2007.05.006 17632043

[B29] Kimmel C. B. Ballard W. W. Kimmel S. R. Ullmann B. Schilling T. F. (1995). Stages of embryonic development of the zebrafish. Dev. Dyn. 203, 253–310. 10.1002/aja.1002030302 8589427

[B27] Knott G. W. Dziegielewska K. M. Habgood M. D. Li Z. S. Saunders N. R. (1997). Albumin transfer across the choroid plexus of South American opossum (*Monodelphis domestica*). J. Physiol. 499(Pt 1), 179–194. 9061648 10.1113/jphysiol.1997.sp021919 PMC1159345

[B51] Kondrychyn I. Garcia-Lecea M. Emelyanov A. Parinov S. Korzh V. (2009). Genome-wide analysis of Tol2 transposon reintegration in zebrafish. BMC Genomics 10:418. 10.1186/1471-2164-10-418 19737393 PMC2753552

[B121] Kondrychyn I. Teh C. Garcia-Lecea M. Guan Y. Kang A. Korzh V. (2011). Zebrafish Enhancer TRAP transgenic line database ZETRAP 2.0. Zebrafish 8, 181–182. 10.1089/zeb.2011.0718 22181660

[B52] Kondrychyn I. Teh C. Sin M. Korzh V. (2013). Stretching morphogenesis of the roof plate and formation of the central canal. PLoS ONE 8:e56219. 10.1371/journal.pone.0056219 23409159 PMC3567028

[B53] Korzh V. (2007). Transposons as tools for enhancer trap screens in vertebrates. Genome Biol. 8(Suppl. 1):S8. 10.1186/gb-2007-8-s1-s8 18047700 PMC2106835

[B31] Korzh V. (2014). Stretching cell morphogenesis during late neurulation and mild neural tube defects. Dev. Growth Differ. 56, 425–433. 10.1111/dgd.12143 24888446

[B28] Korzh V. Edlund T. Thor S. (1993). Zebrafish primary neurons initiate expression of the LIM homeodomain protein Isl-1 at the end of gastrulation. Development 118, 417–425. 8223269 10.1242/dev.118.2.417

[B49] Landsberg R. L. Awatramani R. B. Hunter N. L. Farago A. F. DiPietrantonio H. J. Rodriguez C. I.. (2005). Hindbrain rhombic lip is comprised of discrete progenitor cell populations allocated by Pax6. Neuron 48, 933–947. 10.1016/j.neuron.2005.11.031 16364898

[B10] Lavezzi A. M. Matturri L. Del Corno G. Johanson C. E. (2013). Vulnerability of fourth ventricle choroid plexus in sudden unexplained fetal and infant death syndromes related to smoking mothers. Int. J. Dev. Neurosci. 31, 319–327. 10.1016/j.ijdevneu.2013.04.006 23680292

[B101] Lawson N. D. Weinstein B. M. (2002). *In vivo* imaging of embryonic vascular development using transgenic zebrafish. Dev. Biol. 248, 307–318. 10.1006/dbio.2002.0711 12167406

[B33] Lee K. J. Jessell T. M. (1999). The specification of dorsal cell fates in the vertebrate central nervous system. Annu. Rev. Neurosci. 22, 261–294. 10.1146/annurev.neuro.22.1.261 10202540

[B32] Lee K. J. Dietrich P. Jessell T. M. (2000). Genetic ablation reveals that the roof plate is essential for dorsal interneuron specification. Nature 403, 734–740. 10.1038/35001507 10693795

[B60] Lee Y. N. Shim Y. J. Kang B. H. Park J. J. Min B. H. (2012). Over-expression of human clusterin increases stress resistance and extends lifespan in Drosophila melanogaster. Biochem. Biophys. Res. Commun. 420, 851–856. 10.1016/j.bbrc.2012.03.087 22465014

[B106] Lenard A. Ellertsdottir E. Herwig L. Krudewig A. Sauteur L. Belting H. G.. (2013). *In vivo* analysis reveals a highly stereotypic morphogenetic pathway of vascular anastomosis. Dev. Cell 25, 492–506. 10.1016/j.devcel.2013.05.010 23763948

[B56] Li Y. Allende M. L. Finkelstein R. Weinberg E. S. (1994). Expression of two zebrafish orthodenticle-related genes in the embryonic brain. Mech. Dev. 48, 229–244. 10.1016/0925-4773(94)90062-0 7893604

[B44] Liddelow S. A. Dziegielewska K. M. Vandeberg J. L. Saunders N. R. (2010). Development of the lateral ventricular choroid plexus in a marsupial, *Monodelphis domestica*. Cerebrospinal Fluid Res. 7:16. 10.1186/1743-8454-7-16 20920364 PMC2964622

[B34] Liem K. F. Jr. Tremml G. Jessell T. M. (1997). A role for the roof plate and its resident TGFbeta-related proteins in neuronal patterning in the dorsal spinal cord. Cell 91, 127–138. 10.1016/S0092-8674(01)80015-5 9335341

[B48] Louvi A. Wassef M. (2000). Ectopic engrailed 1 expression in the dorsal midline causes cell death, abnormal differentiation of circumventricular organs and errors in axonal pathfinding. Development 127, 4061–4071. 10952903 10.1242/dev.127.18.4061

[B45] Lowery L. A. Sive H. (2005). Initial formation of zebrafish brain ventricles occurs independently of circulation and requires the nagie oko and snakehead/atp1a1a.1 gene products. Development 132, 2057–2067. 10.1242/dev.01791 15788456

[B81] Luders F. Segawa H. Stein D. Selva E. M. Perrimon N. Turco S. J.. (2003). Slalom encodes an adenosine 3′-phosphate 5′-phosphosulfate transporter essential for development in Drosophila. EMBO J. 22, 3635–3644. 10.1093/emboj/cdg345 12853478 PMC165615

[B85] Luissint A. C. Artus C. Glacial F. Ganeshamoorthy K. Couraud P. O. (2012). Tight junctions at the blood brain barrier: physiological architecture and disease-associated dysregulation. Fluids Barriers CNS 9:23. 10.1186/2045-8118-9-23 23140302 PMC3542074

[B11] Luo X. M. Lin H. Wang W. Geaney M. S. Law L. Wynyard S.. (2013). Recovery of neurological functions in non-human primate model of Parkinson's disease by transplantation of encapsulated neonatal porcine choroid plexus cells. J. Parkinsons Dis. 3, 275–291. 10.3233/JPD-130214 24002224

[B95] Macchi V. Porzionato A. Guidolin D. Parenti A. De Caro R. (2005). Morphogenesis of the posterior inferior cerebellar artery with three-dimensional reconstruction of the late embryonic vertebrobasilar system. Surg. Radiol. Anat. 27, 56–60. 10.1007/s00276-004-0303-6 15645157

[B97] Makanya A. N. Hlushchuk R. Djonov V. G. (2009). Intussusceptive angiogenesis and its role in vascular morphogenesis, patterning, and remodeling. Angiogenesis 12, 113–123. 10.1007/s10456-009-9129-5 19194777

[B35] Millonig J. H. Millen K. J. Hatten M. E. (2000). The mouse Dreher gene Lmx1a controls formation of the roof plate in the vertebrate CNS. Nature 403, 764–769. 10.1038/35001573 10693804

[B117] Mitchell K. Yang H. Y. Berk J. D. Tran J. H. Iadarola M. J. (2009). Monocyte chemoattractant protein-1 in the choroid plexus: a potential link between vascular pro-inflammatory mediators and the CNS during peripheral tissue inflammation. Neuroscience 158, 885–895. 10.1016/j.neuroscience.2008.10.047 19032979 PMC2668531

[B93] Mortazavi M. M. Griessenauer C. J. Adeeb N. Deep A. Bavarsad Shahripour R. Loukas M.. (2014). The choroid plexus: a comprehensive review of its history, anatomy, function, histology, embryology, and surgical considerations. Childs Nerv. Syst. 30, 205–214. 10.1007/s00381-013-2326-y 24287511

[B113] Nataf S. Strazielle N. Hatterer E. Mouchiroud G. Belin M. F. Ghersi-Egea J. F. (2006). Rat choroid plexuses contain myeloid progenitors capable of differentiation toward macrophage or dendritic cell phenotypes. Glia 54, 160–171. 10.1002/glia.20373 16817190

[B4] Netsky M. G. Shuangshoti S. Tennyson V. M. Brightman M. W. Becker N. H. Sutton C. H. (1975). The Choroid Plexus in Health and Disease. Charlottesville, VA: University Press of Virginia.

[B63] Nielsen C. M. Dymecki S. M. (2010). Sonic hedgehog is required for vascular outgrowth in the hindbrain choroid plexus. Dev. Biol. 340, 430–437. 10.1016/j.ydbio.2010.01.032 20123094 PMC2897143

[B66] Nishikawa K. Nakanishi T. Aoki C. Hattori T. Takahashi K. Taniguchi S. (1994). Differential expression of homeobox-containing genes Msx-1 and Msx-2 and homeoprotein Msx-2 expression during chick craniofacial development. Biochem. Mol. Biol. Int. 32, 763–771. 7913646

[B77] Ohto T. Uchida H. Yamazaki H. Keino-Masu K. Matsui A. Masu M. (2002). Identification of a novel nonlysosomal sulphatase expressed in the floor plate, choroid plexus and cartilage. Genes Cells 7, 173–185. 10.1046/j.1356-9597.2001.00502.x 11895481

[B23] Overall M. Bakker M. Spencer J. Parker N. Smith P. Dziadek M. (1997). Genomic imprinting in the rat: linkage of Igf2 and H19 genes and opposite parental allele-specific expression during embryogenesis. Genomics 45, 416–420. 10.1006/geno.1997.4933 9344669

[B54] Papan C. Campos-Ortega J. A. (1999). Region-specific cell clones in the developing spinal cord of the zebrafish. Dev. Genes Evol. 209, 135–144. 10.1007/s004270050237 10079356

[B50] Parinov S. Kondrichin I. Korzh V. Emelyanov A. (2004). Tol2 transposon-mediated enhancer trap to identify developmentally regulated zebrafish genes *in vivo*. Dev. Dyn. 231, 449–459. 10.1002/dvdy.20157 15366023

[B59] Park S. Mathis K. W. Lee I. K. (2014). The physiological roles of apolipoprotein J/clusterin in metabolic and cardiovascular diseases. Rev. Endocr. Metab. Disord. 15, 45–53. 10.1007/s11154-013-9275-3 24097125

[B75] Planas J. V. Capilla E. Gutierrez J. (2000). Molecular identification of a glucose transporter from fish muscle. FEBS Lett. 481, 266–270. 10.1016/S0014-5793(00)02020-2 11007976

[B114] Popescu B. O. Gherghiceanu M. Kostin S. Ceafalan L. Popescu L. M. (2012). Telocytes in meninges and choroid plexus. Neurosci. Lett. 516, 265–269. 10.1016/j.neulet.2012.04.006 22516459

[B57] Power D. M. Elias N. P. Richardson S. J. Mendes J. Soares C. M. Santos C. R. (2000). Evolution of the thyroid hormone-binding protein, transthyretin. Gen. Comp. Endocrinol. 119, 241–255. 10.1006/gcen.2000.7520 11017772

[B78] Ratzka A. Mundlos S. Vortkamp A. (2010). Expression patterns of sulfatase genes in the developing mouse embryo. Dev. Dyn. 239, 1779–1788. 10.1002/dvdy.22294 20503373

[B5] Redzic Z. B. Preston J. E. Duncan J. A. Chodobski A. Szmydynger-Chodobska J. (2005). The choroid plexus-cerebrospinal fluid system: from development to aging. Curr. Top. Dev. Biol. 71, 1–52. 10.1016/S0070-2153(05)71001-2 16344101

[B102] Rehn K. Wong K. S. Balciunas D. Sumanas S. (2011). Zebrafish enhancer trap line recapitulates embryonic aquaporin 1a expression pattern in vascular endothelial cells. Int. J. Dev. Biol. 55, 613–618. 10.1387/ijdb.103249kp 21948709

[B47] Ristoratore F. Carl M. Deschet K. Richard-Parpaillon L. Boujard D. Wittbrodt J.. (1999). The midbrain-hindbrain boundary genetic cascade is activated ectopically in the diencephalon in response to the widespread expression of one of its components, the medaka gene Ol-eng2. Development 126, 3769–3779. 10433907 10.1242/dev.126.17.3769

[B89] Rosenstein J. M. Krum J. M. Sternberger L. A. Pulley M. T. Sternberger N. H. (1992). Immunocytochemical expression of the endothelial barrier antigen (EBA) during brain angiogenesis. Brain Res. Dev. Brain Res. 66, 47–54. 10.1016/0165-3806(92)90138-M 1376220

[B103] Sacilotto N. Monteiro R. Fritzsche M. Becker P. W. Sanchez-Del-Campo L. Liu K.. (2013). Analysis of Dll4 regulation reveals a combinatorial role for Sox and Notch in arterial development. Proc. Natl. Acad. Sci. U.S. A. 110, 11893–11898. 10.1073/pnas.1300805110 23818617 PMC3718163

[B94] Scala G. Corona M. Langella E. Maruccio L. (2011). Microvasculature of the buffalo (*Bubalus bubalis*) choroid plexuses: structural, histochemical, and immunocytochemical study. Microsc. Res. Tech. 74, 67–75. 10.1002/jemt.20875 21181712

[B69] Schmahl J. Raymond C. S. Soriano P. (2007). PDGF signaling specificity is mediated through multiple immediate early genes. Nat. Genet. 39, 52–60. 10.1038/ng1922 17143286

[B108] Scott D. E. Bergevin M. (2005). Fine structural correlates of the choroid plexus of the lateral cerebral ventricle of the human fetal brain. Anat. Rec. 282, 8–12. 10.1002/ar.a.20104 15470665

[B88] Sharifi M. Ciolkowski M. Krajewski P. Ciszek B. (2005). The choroid plexus of the fourth ventricle and its arteries. Folia Morphol. 64, 194–198. 16228955

[B118] Shechter R. Miller O. Yovel G. Rosenzweig N. London A. Ruckh J.. (2013). Recruitment of beneficial M2 macrophages to injured spinal cord is orchestrated by remote brain choroid plexus. Immunity 38, 555–569. 10.1016/j.immuni.2013.02.012 23477737 PMC4115271

[B115] Shuangshoti S. Netsky M. G. (1966). Histogenesis of choroid plexus in man. Am. J. Anat. 118, 283–316. 10.1002/aja.1001180114 5915034

[B12] Spatazza J. Lee H. H. Di Nardo A. A. Tibaldi L. Joliot A. Hensch T. K.. (2013). Choroid-plexus-derived Otx2 homeoprotein constrains adult cortical plasticity. Cell Rep. 3, 1815–1823. 10.1016/j.celrep.2013.05.014 23770240 PMC4119931

[B98] Stainier D. Y. (2001). Zebrafish genetics and vertebrate heart formation. Nat. Rev. Genet. 2, 39–48. 10.1038/35047564 11253067

[B92] Stan R. V. Tse D. Deharvengt S. J. Smits N. C. Xu Y. Luciano M. R.. (2012). The diaphragms of fenestrated endothelia: gatekeepers of vascular permeability and blood composition. Dev. Cell 23, 1203–1218. 10.1016/j.devcel.2012.11.003 23237953 PMC3525343

[B91] Strazielle N. Ghersi-Egea J. F. (2000). Choroid plexus in the central nervous system: biology and physiopathology. J. Neuropathol. Exp. Neurol. 59, 561–574. 10901227 10.1093/jnen/59.7.561

[B6] Strong L. H. (1956). Early development of the ependyma and vascular pattern of the fourth ventricular choroid plexus in the rabbit. Am. J. Anat. 99 249–290. 10.1002/aja.1000990204 13372493

[B120] Szmydynger-Chodobska J. Gandy J. R. Varone A. Shan R. Chodobski A. (2013). Synergistic interactions between cytokines and AVP at the blood-CSF barrier result in increased chemokine production and augmented influx of leukocytes after brain injury. PLoS ONE 8:e79328. 10.1371/journal.pone.0079328 24223928 PMC3815129

[B123] Teh C. Chudakov D. M. Poon K. L. Mamedov I. Z. Sek J. Y. Shidlovsky K.. (2010). Optogenetic *in vivo* cell manipulation in KillerRed-expressing zebrafish transgenics. BMC Dev. Biol. 10:110. 10.1186/1471-213X-10-110 21040591 PMC2989954

[B67] Thisse B. Thisse C. (2005). Functions and regulations of fibroblast growth factor signaling during embryonic development. Dev. Biol. 287, 390–402. 10.1016/j.ydbio.2005.09.011 16216232

[B37] Thomas T. Dziadek M. (1993). Capacity to form choroid plexus-like cells *in vitro* is restricted to specific regions of the mouse neural ectoderm. Development 117, 253–262. 8223250 10.1242/dev.117.1.253

[B104] Trinh le A. Hochgreb T. Graham M. Wu D. Ruf-Zamojski F. Jayasena C. S.. (2011). A versatile gene trap to visualize and interrogate the function of the vertebrate proteome. Genes Dev. 25, 2306–2320. 10.1101/gad.174037.111 22056673 PMC3219234

[B58] Trougakos I. P. (2013). The molecular chaperone apolipoprotein J/clusterin as a sensor of oxidative stress: implications in therapeutic approaches - a mini-review. Gerontology 59, 514–523. 10.1159/000351207 23689375

[B19] Tsuneki K. (1986). A survey of occurrence of about seventeen circumventricular organs in brains of various vertebrates with special reference to lower groups. J. Hirnforsch. 27, 441–470. 3760554

[B65] Tyurina O. V. Guner B. Popova E. Feng J. Schier A. F. Kohtz J. D.. (2005). Zebrafish Gli3 functions as both an activator and a repressor in Hedgehog signaling. Dev. Biol. 277, 537–556. 10.1016/j.ydbio.2004.10.003 15617692

[B22] Umans R. A. Taylor M. R. (2012). Zebrafish as a model to study drug transporters at the blood-brain barrier. Clin. Pharmacol. Ther. 92, 567–570. 10.1038/clpt.2012.168 23047649 PMC5706651

[B26] Venkatesh B. Lee A. P. Ravi V. Maurya A. K. Lian M. M. Swann J. B.. (2014). Elephant shark genome provides unique insights into gnathostome evolution. Nature 505, 174–179. 10.1038/nature12826 24402279 PMC3964593

[B20] Weindl A. Joynt R. J. (1973). Barrier properties of the subcommissural organ. Arch. Neurol. 29, 16–22. 10.1001/archneur.1973.00490250034004 4711802

[B38] Wilting J. Christ B. (1989). An experimental and ultrastructural study on the development of the avian choroid plexus. Cell Tissue Res. 255, 487–494. 10.1007/BF00218783 2706656

[B122] Winata C. L. Kondrychyn I. Kumar V. Srinivasan K. G. Orlov Y. Ravishankar A.. (2013). Genome wide analysis reveals Zic3 interaction with distal regulatory elements of stage specific developmental genes in zebrafish. PLoS Genet. 9:e1003852. 10.1371/journal.pgen.1003852 24204288 PMC3814314

[B119] Wojcik E. Carrithers L. M. Carrithers M. D. (2011). Characterization of epithelial V-like antigen in human choroid plexus epithelial cells: potential role in CNS immune surveillance. Neurosci. Lett. 495, 115–120. 10.1016/j.neulet.2011.03.051 21440040

[B96] Yang J. Dombrowski S. M. Deshpande A. Krajcir N. Luciano M. G. (2010). VEGF/VEGFR-2 changes in frontal cortex, choroid plexus, and CSF after chronic obstructive hydrocephalus. J. Neurol. Sci. 296, 39–46. 10.1016/j.jns.2010.06.012 20619858 PMC2916035

[B8] Zappaterra M. W. Lehtinen M. K. (2012). The cerebrospinal fluid: regulator of neurogenesis, behavior, and beyond. Cell. Mol. Life Sci. 69, 2863–2878. 10.1007/s00018-012-0957-x 22415326 PMC3856656

[B87] Zhang J. Piontek J. Wolburg H. Piehl C. Liss M. Otten C.. (2010). Establishment of a neuroepithelial barrier by Claudin5a is essential for zebrafish brain ventricular lumen expansion. Proc. Natl. Acad. Sci. U.S.A. 107, 1425–1430. 10.1073/pnas.0911996107 20080584 PMC2824400

[B109] Zhang Z. G. Zhang L. Jiang Q. Chopp M. (2002). Bone marrow-derived endothelial progenitor cells participate in cerebral neovascularization after focal cerebral ischemia in the adult mouse. Circ. Res. 90, 284–288. 10.1161/hh0302.104460 11861416

[B105] Zhou T. Wang L. Zhu K. Y. Dong M. Xu P. F. Chen Y.. (2011). Dominant-negative C/ebpalpha and polycomb group protein Bmi1 extend short-lived hematopoietic stem/progenitor cell life span and induce lethal dyserythropoiesis. Blood 118, 3842–3852. 10.1182/blood-2010-12-327908 21828130

